# Corrigendum to “Spinal Antinociceptive Action of Amiloride and Its Interaction with Tizanidine in the Rat Formalin Test”

**DOI:** 10.1155/2016/6435156

**Published:** 2016-09-18

**Authors:** Handong Ouyang, Peizong Wang, Wan Huang, Qian Li, Bilin Nie, Weian Zeng

**Affiliations:** Department of Anesthesiology, State Key Laboratory of Oncology in Southern China, Cancer Center, Sun Yat-Sen University, Collaborative Innovation Center for Cancer Medicine, Guangzhou, China

In this article [1] published in the November/December 2015 issue of the journal, authors Handong Ouyang and Peizong Wang contributed equally to the study.

## References

[1] Ouyang H., Wang P., Huang W., Li Q., Nie B., Zeng W. (2015). Spinal antinociceptive action of amiloride and its interaction with tizanidine in the rat formalin test. *Pain Research and Management*.

